# Information-gathering for self-medication via Eastern Indonesian community pharmacies: a cross-sectional study

**DOI:** 10.1186/s12913-014-0670-6

**Published:** 2015-01-22

**Authors:** Cecilia Brata, Brahmaputra Marjadi, Carl R Schneider, Kevin Murray, Rhonda M Clifford

**Affiliations:** Pharmacy, School of Medicine and Pharmacology, The University of Western Australia, Perth, Australia; Centre of Medicine Information and Pharmaceutical Care, The University of Surabaya, Surabaya, Indonesia; School of Medicine, The University of Western Sydney, Sydney, Australia; Faculty of Pharmacy, The University of Sydney, Sydney, Australia; Centre for Applied Statistics, The University of Western Australia, Perth, Australia

**Keywords:** Information-gathering, Self-medication, Community pharmacies, Indonesia

## Abstract

**Background:**

Gathering sufficient information when handling self-medication requests in community pharmacies is an important factor in assisting patients to obtain appropriate health outcomes. Common types of information usually gathered include patient identity, signs and symptoms, action taken, medical history, and current medications being used. The aims of the study were (1) to describe the types and amount of information gathered by Eastern Indonesian community pharmacy staff when handling self-medication requests, and (2) to identify factors associated with the reported amount of information gathered.

**Methods:**

Patient simulation and pharmacy staff interviews were used. First, patient simulation was conducted using 2 cough scenarios and 1 diarrhoea scenario. Second, a structured interview was administered to eligible pharmacy staff in the setting. The types and amount of information gathered during patient simulation encounters and reported during pharmacy staff interviews were noted. A regression analysis was performed to identify factors associated with the amount of information gathered from the interview data.

**Results:**

The most frequent types of information gathered in patient simulation encounters were the nature of symptoms (88% in one of the cough scenarios) and patient identity (96% in the diarrhoea scenario). Other types of information were gathered in <40% of encounters in each scenario. From the pharmacy staff interviews, >90% of the 173 interviewees reported that they gathered information on patient identity, nature of symptoms, and associated symptoms. Information on medical history and medication used was gathered by 20% and 26% respectively of the 173 interviewees. The majority of pharmacy staff asked 0 to 2 questions in the patient simulation encounters compared to 5 questions pharmacy staff reported as their usual practice during the interviews. Being qualified as a pharmacist or a pharmacy technician was one of the factors positively associated with the reported amount of information gathered.

**Conclusion:**

There were deficits in the types of information gathered when pharmacy staff handling self-medication requests. Having a pharmacy educational background and additional work experience in the pharmacy was positively associated with the reported amount of information gathered. There could be other factors contributing to shortcomings in the actual practice which need to be explored.

## Background

Self-medication, defined as “the selection and use of medicines by individuals to treat self-recognized illnesses or symptoms” [1], is commonly practised all over the world [2-6]. In Indonesia, 91% of the population practised self-medication in 2011 [7], which highlights the important role of community pharmacies that operate as medicine providers, trading under the supervision of formally qualified pharmacists. Instead of merely selling medicines, pharmacists, with their knowledge, can provide consultations for the appropriate use of medicines and build “a responsible self-medication culture” [8].

The provision of self-medication consultations in community pharmacies should consist of patient assessment and the provision of advice [6,9-11]. Patient assessment includes gathering and analysing patient information [6,9,10]. The advice provided may include medical referral, product recommendation, medicine information, non-pharmacological advice, and/or other relevant advice [12].

Gathering adequate information is a crucial component in providing patients with appropriate advice to assist them obtain the most beneficial health outcomes [13]. While there is no standardized protocol of the kinds of information that should be gathered by pharmacists worldwide, a number of common types can be observed in the literature. These include patient identity, signs and symptoms, actions already taken, current medications being used, and medical history [10-12,14,15].

To date, studies of the information-gathering process taking place in Indonesian community pharmacies have focused on metropolitan cities in Western Indonesia [16,17]. There is a paucity of data from the regional areas of Eastern Indonesia which has a different culture and considerably fewer health care resources and facilities [18,19]. Therefore, as part of a larger study to improve pharmacy services for self-medication in this less developed region of Indonesia, we conducted a study to (1) describe the types and amount of information gathered by pharmacy staff when handling self-medication requests, and (2) identify factors that are associated with the reported amount of information gathered.

## Methods

This study was conducted in a provincial capital of Eastern Indonesia. All community pharmacies in 5 sub-districts with high population density in the research area were included. A list of all of these community pharmacies was obtained from the local Department of Health and pharmaceutical wholesalers. Ethical approval was obtained from the Human Research Ethics Committee at the University of Western Australia and the provincial chapter of the Indonesian Pharmacists Association.

In order to obtain comprehensive results, this study utilized a combination of methods: (1) patient simulation and (2) interviews with pharmacy managers and pharmacy staff. The patient simulation and pharmacy staff interview addressed the actual and reported practice respectively. The patient simulation study was undertaken before the interviews.

### Patient simulation

This method used 3 scenarios: 2 scenarios for an Angiotensin-Converting-Enzyme (ACE) inhibitor induced cough (1 symptom-based and 1 product-based) and 1 scenario for acute simple childhood diarrhoea (Table 1). The scenarios and the structured data collection form on the types of information gathered were developed from the literature [10,11,14,15,20,21], and were reviewed by pharmacy academics and practitioners in Indonesia and Australia. A pilot was completed in 10 pharmacies for each cough scenario and 5 pharmacies for the childhood diarrhoea scenario. Data from the pilot were included in the final analysis as there were no changes in the scenario.

**Table 1:** **Description of the simulated patient and the scenarios**

**Symptom-based request (ACE inhibitor induced cough)**	**Product-based request (ACE inhibitor induced cough)**	**Symptom-based request (simple, acute childhood diarrhoea)**
**Description of simulated patients**		
Group 1: A lay person, female, age 60 and a pharmacist, female, age 31	Group 1: A lay person, female, age 60 and a pharmacist, female, age 31	Group 3: A pharmacist, male, age 35 and a pharmacist, female, age 30
Group 2: A lay person, male, age 55 and a pharmacist, male, age 25	Group 2 : A lay person, male, age 55 and a pharmacist, male, age 25	
**Scenario Description**		
On entering the pharmacy, one of the simulated patients said: “What is a good cough medicine that you recommend?”	On entering the pharmacy, one of the simulated patients said: “I want to buy Woods merah^**#**^”	On entering the pharmacy, one of the simulated patients said: “My child has diarrhoea, what do you recommend?”
Information provided (only upon questioning):		Information provided (only upon questioning):
• The patient is the one who has cough. He/she has been coughing for 4 weeks. The cough is dry, irritating and constant. There are no accompanying symptoms.		• The patient is 4 years old, weight ±20 kg, and height: ±1 m.
• The patient tried Bisolvon syrup two weeks ago, but it did not work.		• The patient has acute onset of simple diarrhoea. The diarrhoea started about 6 hours ago. The patient has gone to the toilet three times. The consistency of the stool was mushy, softer than usual.
• The patient was diagnosed with hypertension 2 months ago and routinely consumes captopril 25 mg three times a day.		
• The patient does not have any medical condition other than hypertension and does not routinely consume any medicines, supplements, or herbal medicines other than captopril.		• The patient is generally well, still can play around. Patient is not restless, not irritable, not lethargic, and still has normal drinking habit. The patient has no accompanying symptoms and has not taken any medicines for diarrhoea. The patient has no other medical conditions and does not routinely take any other medications, supplements or herbal medicines. The patient does not have any allergies.
• The patient does not smoke and is not a passive smoker. The patient exercises regularly and follows a healthy diet.		• The patient does not eat anything unusual and no other family members have diarrhoea.
• The blood pressure is controlled (~130/80) and the patient does not have any allergies.		

^**#**^Woods merah is one of the Indonesian brand names of cough medicines that contains Dextromethorphan HBr and Doxylamine.

Six research assistants posed as patients and visited the pharmacies in pairs. The research assistants underwent training on how to enact the scenarios and complete the data collection form. All research assistants signed a confidentiality agreement.

On entering a pharmacy, 1 research assistant of the pair enacted a scenario and purchased any medications recommended by the pharmacy staff while the other person observed the encounter. After each encounter, the simulated patient and the companion independently completed a data collection form, out of sight of pharmacy staff.

The types of information gathered by pharmacy staff in each scenario were analysed descriptively. Inter-rater reliability was measured using the Kappa statistic between the 2 research assistants in each pair.

### Pharmacy staff interviews

The interviews were conducted with 2 types of participants: (a) pharmacy managers and (b) pharmacy staff whose job description included serving patients with self-medication requests.

### Interviews with pharmacy managers

These were face-to-face interviews with the managers of all pharmacies visited in this study. Managers were asked about the characteristics of the pharmacy. The questionnaire comprised of demographic questions regarding the type of pharmacy, the location, the ownership, the service provided, the estimation of total patients served for self-medication and other services per day, the staffing, and the pharmacy opening hours.

### Interviews with eligible pharmacy staff

For participants’ recruitment, the owner or manager of each pharmacy was initially approached for permission to undertake research in their pharmacy and for information about their staff whose job description included serving patients requesting self-medication. No further input was sought from managers who granted permission. One of the researchers (CB) subsequently approached all eligible staff members and asked them to voluntarily participate in the interview. Participants who agreed to participate signed a written informed consent.

The interview was structured and administered by the researcher, who asked about pharmacy staff characteristics and the types and frequency of information gathered when handling a symptom-based self-medication request for a cough. The list of the types of information gathered were developed from the Indonesian pharmacy service standard [20] and other literature (Table 2) [10,11,14,15,21]. A 5-point Likert-type scale was used to reflect the frequency (1 = Never; 2 = Rarely; 3 = Sometimes; 4 = Often; 5 = Always).

**Table 2:** **The types of information gathered (obtained from pharmacy staff interview)**

**The types of information gathered**	**Total number of interviewees that reported gathering the types of information as “often” or “always” (n = 173)**		**Number of interviewees that reported gathering the types of information as “often” or “always” by the professional background**		
			**Pharmacists (n = 42)**	**Pharmacy technicians (n = 32)**	**Staff without formal education in pharmacy (n = 99)**
Patient identity	159 (92%)		41 (98%)	26 (81%)	92 (93%)
Signs and symptoms					
• Nature of symptoms	164 (95%)		42 (100%)	30 (94%)	92 (93%)
• Duration of symptoms	101 (58%)		31 (74%)	15(47%)	55(56%)
• Precipitating factors	38 (22%)		15 (36%)	11(33%)	12 (12%)
• History of the symptoms	30 (17%)		15 (36%)	4 (13%)	11 (11%)
• Accompanying symptoms	162 (94%)		39 (93%)	30 (94%)	93 (94%)
• Danger symptoms*	44 (25%)		16 (28%)	12 (38%)	16 (16%)
Action taken	120 (69%)		37 (88%)	23 (72%)	60 (61%)
Medical history	34 (20%)		11 (26%)	8 (25%)	15 (15%)
Current medications being used	45 (26%)		15 (36%)	7 (22%)	23 (23%)
Allergies	53 (31%)		13 (31%)	12 (38%)	28 (28%)

*Danger symptoms referred to symptoms that warrant medical referral.

The questionnaires used for interviews with pharmacy managers and with eligible pharmacy staff were developed in the Indonesian language by CB, assessed for validity by Australian and Indonesian academics and pharmacy practitioners (English translation was done by bilingual researchers CB and BM); and the final versions were delivered in Indonesian. A pilot was completed by 6 pharmacy managers and 21 pharmacy staff. Only minor changes were effected in the pharmacy staff questionnaire subsequent to the pilot, and therefore all pilot results were included in the analysis.

Test-retest reliability was assessed using the Kappa statistics and the raw percent agreement during the pilot phase. The interval between the test and the retest was 10–14 days. The responses were grouped for Likert scale rating 1 to 3 (never, rarely, and sometimes), and rating 4 to 5 (often and always) for reliability test analysis.

Descriptive analysis was used to summarize data on the characteristics of respondents. Frequencies were calculated for participants who answered “often” or “always” for the type and the amount of information gathered when responding to a symptom-based self-medication request for a cough.

### Data analysis for factors associated with the reported amount of information gathered

Given that data originated from multiple staff per pharmacy, univariable and multivariable analyses using ordinal logistic regression with generalized estimating equations were used to identify factors associated with the amount of information gathered from interview data. The amount of information gathered was used as the dependent variable since previous studies have found that a greater amount of information gathered is associated with the likelihood of providing appropriate advice [22,23]. The frequency of asking information as stated in Table 2 was used to categorize respondents into 3 groups: “low” (0–3 types of information were reported as “often” or “always”), “medium” (4 to 7 types of information), and “high” (8 to 11 types of information). Independent variables tested from pharmacy characteristics were: the types of pharmacy; the number of patients served per day; the number of patients requesting self-medication per day; total pharmacists’ working hours per week; and total pharmacy technicians’ working hours per week. Independent variables tested from pharmacy staff characteristics were: professional background (i.e., pharmacists, pharmacy technicians, or staff without formal education in pharmacy); age; gender; work experience; and participation in any post-graduation training on self-medication. Respondents’ age was dichotomized around the mean (<30 years old and ≥30 years old), while work experience was categorized based on the median (≤3 years and >3 years). Test of parallel lines was undertaken to test the proportional odds assumption. Variables were considered statistically significant and retained in the final multivariate model where p < 0.05. Odds ratios (OR), 95% confidence intervals (CI), and p values were reported. IBM SPSS Statistics for Macintosh version 22 (Armonk, NY: IBM Corp) was used for the analysis.

## Results

### Study participants

#### Patient simulation method

The total population of pharmacies was 78 for the cough scenarios and 81 for the childhood diarrhoea scenario. Differences in the total population of pharmacies were due to 3 new pharmacies commencing business between data collection periods. Data collection was conducted at 2 different times. The first data collection, using the cough scenarios, was conducted from June 2011 to August 2011, and the second data collection, using the diarrhoea scenario, was conducted in May 2012. Of the 78 pharmacies, 76 (97%) and 69 (88%) pharmacies were visited for the symptom-based request and the product-based request for cough respectively. Of the 81 pharmacies eligible for the childhood diarrhoea scenario, 80 (99%) pharmacies were visited. In total, there were 12 visits for the 3 scenarios that were not made. This was because the pharmacies were closed at the time of visitation or because the simulated patients recognized the pharmacy staff on duty.

#### Pharmacy manager interviews

**Figure 1:**
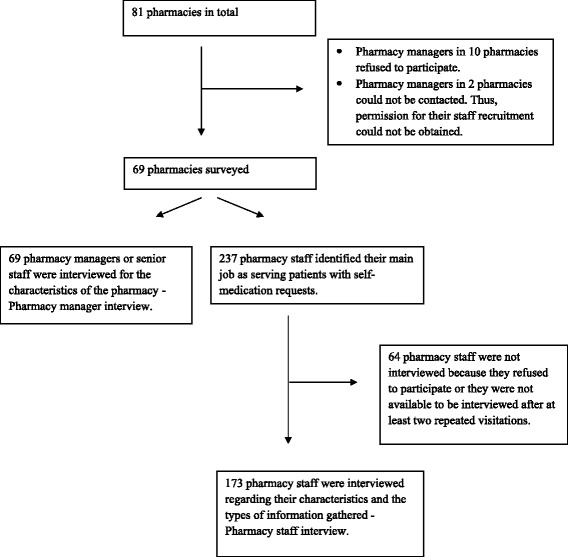
**Study participants for pharmacy manager and pharmacy staff interviews.**

The total population of pharmacies was 81 at the time of data collection. Sixty-nine of 81 pharmacy managers (85%) were interviewed (Figure 1). These interviews were conducted simultaneously with pharmacy staff interviews after the patient simulation study had been completed.

#### Pharmacy staff interviews

There were 173 pharmacy staff from 69 pharmacies interviewed (Figure 1). These included 42 pharmacists, 32 pharmacy technicians, and 99 staff without formal pharmacy education.

### The reliability results

The Kappa scores from the simulated patients ranged from 0.89 to 1, which indicated very good reliability [24]. From the pharmacy staff interviews, the test-retest reliability showed Kappa scores that ranged from 0.21 to 0.86 (fair to very good reliability) [24], and raw percent agreement that ranged from 71% to 95%. It is noticeable that many responses were clustered on either the higher or the lower group, which could lead to an arbitrarily low Kappa [25].

### Pharmacy characteristics

The characteristics of pharmacies obtained from the pharmacy managers interviews are presented in Table 3. The services provided were basic, including services for self-medication (100%) and prescription medication (>90%). An estimated 65% of patients visited the pharmacies for self-medication requests. The total number of staff employed in the 69 pharmacies surveyed was 352; the majority of them (54%) did not have any formal education in pharmacy. The majority of pharmacist managers (80%) had primary jobs as government employees. To estimate the availability of pharmacists and pharmacy technicians in each pharmacy, a ratio of total working hours of each profession per week to total pharmacy opening hours per week was calculated. The results showed a ratio between 0 and 1.05 for pharmacists with a median of 0.1 and inter-quartile range of 0 to 0.3. Of the 69 pharmacies surveyed, 99% had a ratio less than 1 for pharmacists’ availability. The results for pharmacy technicians showed a ratio between 0 and 2.4 with a median of 0.5 and inter-quartile range of 0.1 to 1.05. Of the 69 pharmacies surveyed, 75% had a ratio less than 1 for pharmacy technicians’ availability. This indicated that in many pharmacies, either pharmacists or pharmacy technicians were not always available during pharmacy opening hours.

**Table 3:** **Pharmacy characteristics**

**Pharmacy characteristics**	**n = 69 (%)**
Pharmacy type	
• Attached to doctor’s clinic	50 (72%)
• Not attached to doctor’s clinic	19 (28%)
Pharmacy location	
• Street	62 (90%)
• Residential areas	3 (4%)
• Traditional market	3 (4%)
• Shopping mall	1 (1%)
Pharmacy ownership	
• Pharmacist	7 (10%)
• Non-pharmacist	60 (87%)
• Other (i.e., joint business of pharmacist and non-pharmacist)	2 (3%)
Pharmacy services	
• Self-medication services	69 (100%)
• Compounding prescription medication services	65 (94%)
• Non-compounding prescription medication services	68 (99%)
• Other (e.g., health promotion, etc.)	2 (3%)
Estimated total patients served per day (from 67 pharmacies)*	4595
Estimated total patients served for self-medication per day (from 67 pharmacies)*	2975
Total staff employed in the 69 pharmacies surveyed	352
• Total number of pharmacists	75
• Total number of pharmacy technicians	86
• Total number of staff who do not have formal education in pharmacy	191
Total staff working hours per week in the 69 pharmacies surveyed	
• Pharmacists	1270
• Pharmacy technicians	3542
• Other staff who do not have formal education in pharmacy	8973
Total pharmacy opening hours per week in the 69 pharmacies surveyed	6198
Number of pharmacies which had a ratio of pharmacists’ working hours per week to total pharmacy opening hours as <1**	68 (99%)
Number of pharmacies which had a ratio of pharmacy technicians’ working hours per week to total pharmacy opening hours as <1**	52 (75%)
Number of pharmacies in which the pharmacist manager had a primary job other than being a pharmacy manager (i.e., as a government employee)	55 (80%)

*Calculated from 67 pharmacies. There were 2 missing data for these questions.

**Ratio <1 implied that pharmacists or pharmacy technicians were not always available during pharmacy opening hours.

### Pharmacy staff characteristics

The majority of respondents whose job description included serving patients with self-medication requests were female (81%) with a mean age of 30 years (Table 4). Fifty-seven percent of these respondents (99/173) did not have any formal education in pharmacy. Only 19 of 173 interviewees (consisting of 9 pharmacists, 2 pharmacy technicians, and 8 staff without formal pharmacy education) ever attended any self-medication training after attaining their highest education qualification.

**Table 4:** **Pharmacy staff characteristics**

**Pharmacy staff characteristics**	**n = 173 (%)**
Professional background	
• Pharmacists^$^	42 (24%)
• Pharmacy technician*	32 (19%)
• Staff without formal educational background in pharmacy	99 (57%)
Age (years; mean ± SD)	30 ± 7.8
Gender : female	140 (81%)
Highest education qualification	
• Pharmacist registration training program	42 (24%)
• Bachelor degree in pharmacy	2 (1%)
• Three year diploma in pharmacy	21 (12%)
• Pharmacy assistant school equivalent to senior high school	9 (5%)
• Senior high school	79 (46%)
• Others (i.e., bachelor degree or diploma in subjects other than pharmacy)	20 (12%)
Working experience (years; median, IQR^#^)	3, IQR = 1.5 – 7
Ever attended training on self-medication after graduation from the highest education qualification	
• Yes	19 (11%)
• No	154 (89%)

^$^A pharmacist in Indonesia is a person who has a bachelor degree in pharmacy and holds a pharmacist registration training certificate.

*A pharmacy technician in Indonesia is a person who has graduated from pharmacy assistant school or has three year diploma in pharmacy or a person who has a bachelor degree in pharmacy without holding a pharmacist registration training certificate.

^#^IQR = Interquartile range.

### The types of information gathered

#### Patient simulation results

Information was gathered in 99% of symptom-based self-medication requests for cough, 96% of symptom-based self-medication requests for diarrhoea, and only 9% of product-based self-medication request for cough. The most common types of information gathered by pharmacy staff was the nature of cough in the symptom-based cough scenario (88% of 76 encounters) and patient identity for the diarrhoea scenario (96% of 80 encounters, see Table 5). Information on medical history was gathered in only 4% of encounters using the symptom-based cough scenario and 0% in the other scenarios. Information on medication used was not gathered in any encounters.

**Table 5:** **The types of information gathered (obtained from patient simulation)**

**The types of information gathered**	**Symptom-based requests (ACE inhibitor induced cough) n = 76 pharmacies**	**Product-based request (ACE inhibitor induced cough) n = 69 pharmacies**	**Symptom-based request (Simple, acute childhood diarrhoea) n = 80 pharmacies**
• Patient identity	23 (30%)	2 (3%)	77 (96%)
• Signs and symptoms, This included:			
o Nature of symptoms (i.e., whether the cough productive or non productive in the 2 cough scenarios; and stool consistency and frequency of diarrhoea in the childhood diarrhoea scenario).	67 (88%)	4 (6%)	22 (28%)
o Duration of symptoms	16 (21%)	0 (0%)	27 (34%)
o History of symptoms	3 (4%)	0 (0%)	0 (0%)
o Precipitating factors	0 (0%)	0 (0%)	4 (5%)
o Accompanying symptoms	28 (37%)	0 (0%)	12 (15%)
o Danger symptoms*	0 (0%)	0 (0%)	0 (0%)
• Action taken	12 (16%)	0 (0%)	5 (6%)
• Medical history	3 (4%)	0 (0%)	0 (0%)
• Current medications being used	0 (0%)	0 (0%)	0 (0%)
• Allergies	0 (0%)	0 (0%)	0 (0%)

*Danger symptoms are defined as symptoms that warrant medical referral. For the cough scenarios, danger symptoms constituted symptoms such as chest pain, shortness of breath, wheezing, green/rusty/blood in sputum. For the diarrhoea scenarios, danger symptoms constituted symptoms such as blood/mucus in the stool and severe dehydration signs.

#### Pharmacy staff interview results

Information on patient identity, nature of symptoms, and associated symptoms was reportedly gathered by >90% of the 173 interviewees (Table 2). Information on medical history and medication used was reported to be gathered by 20% and 26% respectively of the 173 interviewees. These information types were rarely gathered by interviewees regardless of their educational background.

### The amount of information gathered

#### Patient simulation results

**Figure 2:**
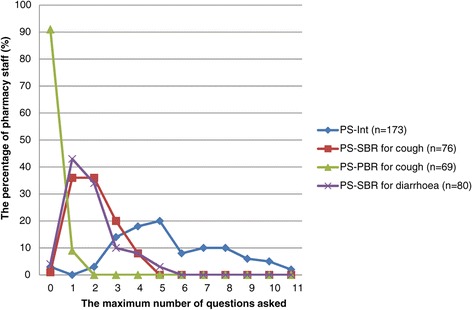
**The amount of information gathered.** PS-Int: Pharmacy staff interview results (n = 173); PS-SBR for cough: Patient simulation results of symptom-based self-medication requests for cough (n = 76); PS-PBR for cough: Patient simulation results of product-based self-medication requests for cough (n = 69); PS-SBR for diarrhoea: Patient simulation results of symptom-based self-medication requests for childhood diarrhoea (n = 80).

For symptom-based self-medication requests for cough, pharmacy staff in 1% of 76 encounters did not ask any questions and the maximum number of questions asked was 4. For product-based self-medication requests for cough, pharmacy staff in 91% of 69 encounters did not gather any information and the maximum number of questions asked was only 1. For the diarrhoea scenario, no information was gathered in 4% of 81 encounters and the maximum numbers of questions asked was 5 (Figure 2).

#### Pharmacy staff interview results

The majority of pharmacy staff (53%; 91/173 interviewees) reported the frequency of asking 3 to 5 questions as “often” or “always” when presented with symptom-based self-medication requests for cough (Figure 2).

### Factors associated with the amount of information gathered

Univariable analysis from pharmacy staff interviews identified that being a pharmacist or a pharmacy technician as opposed to a staff member without formal education in pharmacy, work experience of more than 3 years, and total pharmacist working hours per week are factors that associated positively with gathering more information (Table 6). After including all factors, multivariable analysis identified that being a pharmacist or a pharmacy technician as opposed to a staff member without formal education in pharmacy, work experience of more than 3 years, age below 30 years, and total pharmacists working hours per week were associated positively with the amount of information gathered.

**Table 6:** **Factors associated with the reported amount of information gathered**

**Factors**	**Univariable analysis**		Multivariable analysis*	
	**OR (95% CI)**	**P value**	**OR (95% CI)**	**P value**
Professional background				
• Pharmacist	**3.0 (1.64 – 5.42)**	**<0.001**	**2.4 (1.29 – 5.44)**	**0.006**
• Pharmacy technician	**2.6 (1.06 – 6.35)**	**0.038**	**3.0 (1.10 – 8.00)**	**0.031**
• Staff without formal education in pharmacy	Reference		Reference	
Age				
• <30 years old	1.3 (0.74-2.26)	0.370	**2.4 (1.21 – 4.65)**	**0.012**
• ≥30 years old	Reference		Reference	
Gender				
• Male	0.99 (0.50 – 1.98)	0.984		
• Female	Reference			
Working experience				
• ≤3 years	**0.4 (0.23-0.75)**	**0.004**	**0.3 (0.14 – 0.50)**	**<0.001**
• >3 years	Reference		Reference	
Post-graduation training in self-medication				
• Yes	2.2 (0.84 – 5.75)	0.109		
• No	Reference			
Type of pharmacy				
• Attached to doctors’ clinic	2.0 (0.99 – 4.03)	0.055		
• Not attached to doctors’ clinic	Reference			
Total number of patients served per day.	1.0 (0.99, 1.01)	0.867		
Total number of patients requesting self-medication served per day.	1.0 (0.99, 1.01)	0.893		
Total pharmacists’ working hours per week.	**1.02 (1.01 – 1.03)**	**0.005**	**1.02 (1.00 – 1.03)**	**0.011**
Total pharmacy technicians’ working hours per week.	1.0 (0.99 – 1.01)	0.999		

Bold value indicated variables that were significantly associated with an increase in the amount of information gathered based on p < 0.05.

*Only significant variables were presented.

## Discussion

Information-gathering is important for ensuring optimal patient outcomes [6]. Our study found more favourable results from interview data (“reported practice”) than patient simulation data (“actual practice”). For example, the majority of pharmacy staff only asked 1 or 2 questions in actual practice as compared to 5 questions according to their reported practice. Discrepancies between the actual and reported practice have been reported [26-28]. The discrepancies between reported and actual practices might be explained by either or both of 2 principles, namely: social desirability bias and the phenomenon of intention not translated into action. Social desirability bias is defined as “the tendency of some respondents to report an answer in a way they deem to be more socially acceptable than would be their true answer” [29]. The second view to explain the discrepancy between reported and actual practice is a phenomenon where intention does not always translate into action [30,31]. In this case, the pharmacy staff interviews can be regarded as a measure of intention while patient simulation is a measure of action. While intention is usually strongly related to action, there are cases where there are factors that may hinder the implementation of intention into action [31]; these factors are still yet to be researched in this setting.

Although our study showed that results from the reported practice are more favourable than the actual practice, both methods showed deficits in the information-gathering process, particularly in the types of information gathered. Information such as current medication being used and medical history was rarely gathered in both patient simulation encounters and pharmacy staff interviews. This phenomenon has been consistently seen in other studies conducted in developing countries [32]. Deficits in information-gathering may result in inappropriate advice being provided [22], which can lead to a delay in medical treatment and irrational use of medicines, thereby resulting in a poor patient outcome [33,34].

In the patient simulation scenarios of ACE inhibitor induced cough, none of the pharmacy staff asked about the current medications being used by the simulated patient. As a result, pharmacy staff failed to detect the underlying problem of the cough for this scenario. In the childhood diarrhoea scenario, information was rarely sought to determine the existence and degree of dehydration. Complete information regarding the symptoms was also rarely asked by pharmacy staff. Without this information, it would be difficult for pharmacy staff to manage this diarrhoea case appropriately. Our study has shown that pharmacy staff’s performance of information-gathering for self-medication requests needs to be improved.

Different types of requests have an influence on the information-gathering process. In our patient simulation study, the proportion of pharmacy staff gathering at least one type of information was considerably higher for symptom-based self-medication requests (98%); compared to product-based self-medication requests (9%). This is consistent with other research [23,35,36]. Patients’ familiarity with the use of the product and patient resistance to questioning have been postulated as factors that may cause less information to be gathered in product-based self-medication requests [23,36]. However, it is important that pharmacy staff still take an adequate history so that the product requested is appropriate for the patient’s condition. Without gathering adequate information, a community pharmacy does not have the added value of a health care provider, but is little more than business entity akin to a supermarket or other retail outlets [36].

Qualified pharmacists or pharmacy technicians, and pharmacies which had higher pharmacists’ working hours per week, were shown to be positively associated with the reported amount of information gathered (Table 6). The formal training of pharmacists and pharmacy technicians might provide some basic knowledge of gathering information from patients. Other factors were having work experience of more than 3 years and being aged below 30 years. It could be that with more years of experience, pharmacy staff may gain more knowledge of the type of questions which should be asked. Meanwhile, positive results between younger staff and the reported amount of information gathered might be due to step-by-step changes in the Indonesian pharmacy curriculum during the last 10 years, previously focusing on pharmaceutical science, into a curriculum focusing on pharmacy practice/clinical pharmacy [37]. The factors identified from our statistical analysis, however, were associated with the reported practice of pharmacy staff (that is the intentions of pharmacy staff). There could be factors other than intention that may influence the actual practice which are yet to be researched. Qualitative approach may be more suitable for exploring factors influencing actual practice in this setting.

The reasons of inadequate practice are often complex and multi-factorial and need to be considered in context [38-40]. In Indonesia, the trained pharmacy workforce includes pharmacists and pharmacy technicians. Pharmacists need to complete 5 years of training, while pharmacy technicians need to complete 3 or 4 years of education in pharmacy [41]. Local data from the province where this study was conducted showed deficiencies in the number of trained pharmacists and technicians in the workforce [42]. Because of this, trained pharmacy staff usually work as full-time employees in the public sector and work part time in community pharmacies, leading to low availability of trained pharmacy staff in community pharmacies (Table 3). This situation can cause inadequate supervision of the non-trained workforce in community pharmacies and may lead to inadequate performance when handling self-medication requests. In addition, there could be variations of how the topic of self-medication consultation is taught and assessed in Indonesian pharmacy schools [43-46]. As a consequence, graduates’ knowledge and skills in relation to self-medication consultation may also vary. Further research focusing on pharmacy education for self-medication consultation and standardization of curriculum and assessment for self-medication topic are needed.

The reasons for the poor performance in information-gathering by pharmacy staff can be very complex and not simply a result of pharmacy staff failing to perform their required duties. There could be other socio-cultural contextual factors that have not been discussed and still need to be researched. It is suggested that future research could focus on identifying and addressing the many barriers that hinder pharmacy staff performing appropriate information-gathering when responding to self-medication requests in this population.

### Strengths and limitations

To our knowledge, this was the first study examining the information-gathering performed by pharmacy staff when handling self-medication requests in a less developed region in Indonesia. The use of 2 methods gave a complete picture of the information-gathering practice of pharmacy staff (actual and reported).

There were limitations in our study. Only one visit per scenario was made in the patient simulation study and thus it may not provide an accurate picture of the actual practice of each pharmacy staff member. However, the consistency of the findings can provide an overall picture of information-gathering performed by pharmacy staff when handling self-medication requests in this setting. Furthermore, both of the patient simulation and pharmacy staff interview studies were confined to a limited number of specific scenarios and thus the findings may not be generalized to other scenarios. The results, however, consistently showed insufficient types of information gathered in all scenarios used.

## Conclusion

While more favourable results were found from interview data (“reported practice”) than patient simulation data (“actual practice”), both studies showed deficits in the types of information gathered when handling self-medication requests. This may lead to inappropriate advice being provided by pharmacy staff and put patients at risk of using medicines inappropriately.

Staff who had an educational background in pharmacy and additional years of work experience were positively associated with the reported amount of information gathered. There could be other factors that influence insufficient information-gathering in the actual practice in this study population. Therefore, further research is suggested to explore the reasons for insufficient information-gathering in this study population.
